# Calcaneal Lengthening after Tarsal Bone Fusion for Massive Calcaneus Defect Reconstruction

**DOI:** 10.1111/os.14230

**Published:** 2024-09-05

**Authors:** Rui Zhang, Xiangyun Yao, Xiaoyu Wang, Xu Zheng, Haoran Mu, Hongjiang Ruan, Qinglin Kang

**Affiliations:** ^1^ Department of Orthopedics Shanghai Sixth People's Hospital Affiliated to Shanghai Jiao Tong University School of Medicine Shanghai China; ^2^ Department of Orthopedics Shanghai Bone Tumor Institution, Shanghai General Hospital, Shanghai Jiao Tong University School of Medicine Shanghai China

**Keywords:** Calcaneus Defect, Foot and Ankle, Function, Lengthening, Reconstruction

## Abstract

**Objectives:**

Calcaneus defect remains challenging with limited strategies for reconstruction. Current methods, including graft transplantation, substitution, and distraction osteogenesis, showed limited advantages with certain shortcomings. Current calcaneus lengthening for partial calcaneus loss reconstruction requires bone loss of less than 35%. We introduced our combination of tarsal bone fusion and gradual lengthening method in treating massive calcaneus loss.

**Methods:**

From January 2015 to December 2021, tarsal bone fusion and calcaneus gradual lengthening were performed in six patients with unilateral massive traumatic loss of the calcaneal tuberosity. A retrospective study was held to evaluate the outcomes of this novel technique. Clinical outcomes were assessed based on the American Orthopedic Foot and Ankle Score (AOFAS). Radiological data were assessed, which included tibio‐calcaneal angle (TCA), calcaneal interface angle (CIA), metatarsal declination angle (MDA), angle of longitudinal arch (ALA), and the amount of calcaneus axial lengthening (CAL).

**Results:**

The mean calcaneal axial lengthening was 43.8 ± 3.1 mm (range, 39–49.5 mm), and the mean proportion of the lengthened calcaneus was 47.8% ± 3.7% (range, 42.8–55.3%). The mean external fixation time was 104.8 ± 67.5 days (range, 69 to 242 days), and the mean external fixation index was 2.4 ± 1.6 days/cm. All patients stuck to the postoperative follow‐up plan with an average follow‐up time (FT) of 35.0 ± 6.7 months (range, 26–40 months). Deformities of the injured limbs were all corrected according to radiography. Based on the AOFAS, three excellent and three good results were achieved.

**Conclusion:**

The Ilizarov technique remains an option for calcaneus reconstruction with a great amount of loss once combined with tarsal bone fusion. The function of the injured foot and ankle can be satisfactorily restored using these techniques in our study. Apart from calcaneus elongation, tarsal bone fusion is somehow necessary to reinforce the proximal segment of the distracted calcaneus for creating a larger distraction callus, correcting concomitant foot deformities, and enhancing hindfoot stability. It is necessary to choose flexibly when tarsal bones should be fused.

## Graphical abstract

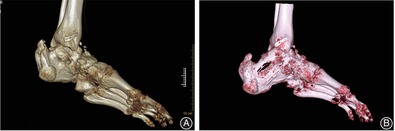

## Introduction

Partial or total loss of the calcaneus, which is the crucial bony component of the hindfoot, will significantly impair lower limb weight‐bearing function, leading to family and socioeconomic burdens.^1^, ^2^

Different kinds of autografts, allografts, and prostheses have been introduced for calcaneus defect reconstruction.^3^, ^4^ Although these approaches can restore the structure and function of the damaged hindfoot, their efficacy is somehow limited due to prolonged time for microsurgical anastomosis, donor‐site morbidity, bone resorption, poor biocompatibility, and material fatigue, and below‐knee amputation is required in some severe cases.^3^

Calcaneus with partial defect, as well as its surrounding tissues, can be gradually lengthened through distraction histogenesis, which represents an alternative method for limb salvage in such cases. However, it has been emphasized that the amount of calcaneus defect should not exceed 35% when applying the Ilizarov technique in calcaneus reconstruction; otherwise, adequate fixation of calcaneal segments and safe calcaneal osteotomy might not be obtained.^4^ On the contrary, arthrodesis is usually performed to alleviate terminal joint pain and correct limb alignment, especially in the field of foot and ankle surgery. Whether a combination of hindfoot joint fusion and calcaneus elongation would break through the upper limit of 35% loss of calcaneus for its defect reconstruction has never been reported in previous studies.

In this retrospective study, we (i) put forward our experience in treating massive calcaneus defects over 35% of the healthy calcaneus side with the combination of tarsal bone fusion and gradual lengthening and (ii) evaluated functional and radiological outcomes of these patients.

## Patients and Methods

### Ethics Approval

The study protocol conforms to the ethical guidelines of the Declaration of Helsinki and was approved by our Hospital Ethics Committee (No. 2023‐KY‐051(k)). The patients and/or their relatives were informed that their clinical data would be collected for publication, and we received their consent.

### Basic Information and Inclusion/Exclusion Criteria

From January 2015 to December 2021, tarsal bone fusion and calcaneus lengthening were performed in six patients who suffered from unilateral massive traumatic loss of the calcaneal tuberosity. Preoperative two‐dimensional radiographs and computed tomography images with three‐dimensional reconstruction of the bilateral foot and ankle were obtained in all patients for a better understanding of the deformity and surgical planning.

Patients' data were included when^1^ their age range was between 18 and 60 years,^2^ the amount of calcaneus loss was between 35% and 75%,^3^ the talocalcaneal joint line was intact, or the patient received previous subtalar arthrodesis,^4^ hindfoot soft tissue coverage had been accomplished, and^5^ received our first‐stage calcaneus lengthening and tarsal bone fusion and second‐stage screw fixation for external frame removal. Patients^1^ who refused surgical reconstruction,^2^ whose calcaneus defect could not allow external frame fixation or could be elongated immediately,^3^ whose talocalcaneal joint line was not intact,^4^ who had associated infection or on‐set severe mental diseases, diabetes, gout, and rheumatoid arthritis, or^5^ whose calcaneal defect was caused by other diseases; for example, congenital atrophic calcaneus and calcaneal malignancies were excluded. The amount of calcaneus loss was measured using the method introduced by Wang et al.^4^

Surgical indications are that^1^ the amount of calcaneus loss was between 35% and 75%,^2^ the talocalcaneal joint line should be intact, or the patient received previous subtalar arthrodesis,^3^ hindfoot soft tissue coverage had been accomplished, and^4^ the patient's willingness for surgical reconstruction.

### Surgical Procedures and Postoperative Care

The same senior surgeon performed all operations. Two stages of operations were performed.

#### First‐Stage Operation and Postoperative Management

The first operation was carried out under general anesthesia in all patients. First, immediate Achilles tendon lengthening and tenolysis were performed, and then the tibialis anterior muscle was explored and halved, the lateral part of which was anchored to the cubitus bone. Calcaneocuboid arthrodesis, subtalar arthrodesis, and talonavicular arthrodesis were also carried out. Intraoperative fluoroscopy was applied to check the correction of hindfoot varus. The plantar fascia was severed to expose the residual of the calcaneus. Additional Kirschner wires were applied for tarsometatarsal fixation and calcaneocuboid fixation.

The external fixator was then constructed, and calcaneal osteotomy was accomplished under fluoroscopic guidance. The bone cut should not interfere with the fused subtalar joint. All incisions were precisely washed and sutured before the bind‐up.

Gradual distraction began at a rate of 0.75 mm per day after a latency of 7–10 days. During the lengthening period, the remnant of calcaneal tuberosity was pulled to the inferior and posterior foot. All patients stuck to the follow‐up schedule, which included monthly outpatient appointments until the angle of a longitudinal arch (ALA) of the foot reached from 125° to 130°. The axial lengthening was stopped and horizontal lengthening was then performed to enhance the capability against yield failure of the reconstructed calcaneal body.

#### Second‐Stage Operation and Postoperative Management

Screw fixation and removal of the external fixators were performed in the second‐stage operation once the calcaneus was lengthened equal to the contralateral one.

Two anti‐rotation screws were inserted through the transferred calcaneal tuberosity for callus fixation and subtalar stabilization before the external fixators were removed. The two screws were removed when bone consolidation was confirmed via fluoroscopy.

Patients were encouraged to activate the ipsilateral hip, knee, and ankle and to bear partial weight at the forefoot to prevent disuse osteoporosis, tendon adhesion, and joint stiffness. Full weigh‐bearing was allowed 6 weeks after screw fixation. All patients kept follow‐up after the removal of the screws to check lower limb appearance, function, and complications.

### Data Collection

Demographic data, including age, gender, side of the defect, trauma‐to‐calcaneus‐reconstruction interval (TCRI), fFT, and external fixation‐related data such as external fixator time (EFT) and external fixator index (EFI) were collected via electric medical history system. EFI was calculated through the following formulas:

$$\mathrm{EFI}=\frac{\mathrm{EFT}}{\mathrm{CAL}}\;\left(\text{days}/\mathrm{cm}\right).$$

**FIGURE 1:**
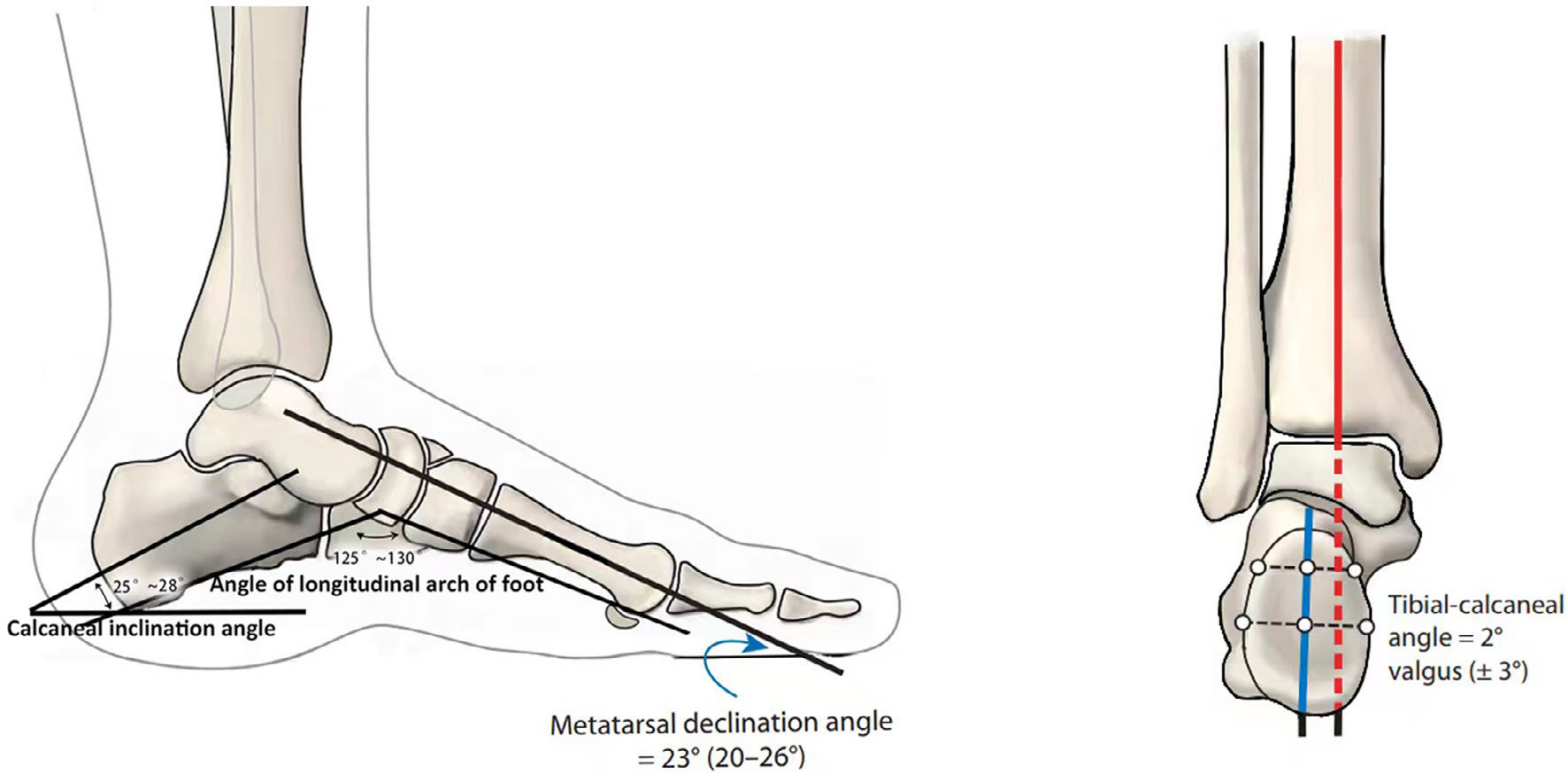
Angular indices of the affected foot and ankle were measured on different views. Calcaneal inclination angle refers to the angle between the calcaneal axis and the horizon on the lateral view of the foot. The angle of a longitudinal arch (ALA) of the foot refers to the intersection between the tangent line of the inferior border of the calcaneus and the tangent line of the inferior border of the first metatarsal bone on the lateral view of the foot. Metatarsal declination angle (MDA) refers to the angle between the axis of the first metatarsal bone and the horizon on the lateral view. Tibio‐calcaneal angle (TCA) refers to the intersection between the anatomic axis of the distal tibia and that of the calcaneus on the anteroposterior view of the ankle. All normal ranges of these angular indices were shown.

We evaluated the range of motion (ROM) of the ankle preoperatively and at the time of the final follow‐up with a goniometer. Single‐leg heel lift (HL) was also checked at the same time points. Calf circumference (CC) and HL were measured to determine muscle wasting of the ipsilateral lower leg.^5^ Radiological data were assessed, which included tibio‐calcaneal angle (TCA), calcaneal interface angle (CIA), metatarsal declination angle (MDA), ALA, and the amount of calcaneus axial lengthening (CAL) (Figure 1).

Subjective satisfaction of each patient was reported using a 100‐mm visual analog scale (VAS, 0 mm equals “totally unsatisfied” and 100 mm equals “completely satisfied”).^6^ Symptom Checklist‐90‐Revised (SCL‐90‐R), a brief self‐report psychometric questionnaire, was also applied to evaluate patients' psychological situation.^7^ Outcomes were assessed using the American Orthopedic Foot and Ankle score (AOFAS) as poor (0–50), fair (50–74), good (75–90), and excellent (90–100).^8^

### Statistic Analysis

Statistic analysis was performed with GraphPad Prism 8.0 (Graphpad Software, Inc., San Diego, CA, USA). Continuous variables were presented as ^−^ *x* ± *s*. A paired two‐group *t*‐test was applied to compare ROM, ALA, TCA, CIA, MDA, CAL, VAS, SCL‐90‐R, and AOFAS previously and at the last follow‐up. A *p* value less than 0.05 was considered a significant difference.

## Result

### Demographic Data

Five male patients and one female patient were included with an average age of 22.3 ± 3.0 years (range, 19–26 years). Three of them were injured due to falling accidents, and the rest due to traffic accidents. All injuries were unilateral. Soft tissue reconstruction was accomplished by the former surgical team with only calcaneus defect left. The mean TCRI was 24.0 ± 6.4 months (range, 17–34 months), a rather prolonged interval due to complicated management and rehabilitation after the initial attack. The mean CAL was 43.8 ± 3.1 mm (range, 39–49.5 mm), and the mean proportion of the lengthened calcaneus was 47.8 ± 3.7% (range, 42.8%–55.3%). The mean EFT and EFI were 104.8 ± 67.5 days (range, 69–242 days) and 2.4 ± 1.6 days/cm, respectively. Delayed removal of the external fixation was performed in one patient because of the local coronavirus disease 2019 (COVID‐19) lockdown policy. All patients stuck to the postoperative follow‐up plan with an average FT of 35.0 ± 6.7 months (range, 26–40 months). No major complications were observed (Table 1).

**TABLE 1:** Demographic data.

Case	Age (years)	Gender	Side	Cause	TCRI (months)	FT (months)	CAL (mm/%)	EFT (days)	EFI (days/mm)
1	20	Male	Left	FA	23	26	42.0/44.9	242	5.76
2	24	Male	Right	FA	17	40	49.5/53.2	83	1.68
3	19	Male	Left	FA	28	37	39.0/42.9	69	1.77
4	26	Male	Left	TA	34	43	45.0/47.1	84	1.87
5	20	Female	Right	TA	18	28	41.0/50.0	69	1.68
6	25	Male	Left	TA	24	36	46.0/48.9	82	1.78

Abbreviations: CAL, calcaneus axial lengthening (extra length/its proportion of the healthy calcaneus); EFI, external fixation index; EFT, external fixation time; FA, falling accident; FT, follow‐up time; mm, milimeter; TA, traffic accident; TCRI, trauma‐to‐calcaneus‐reconstruction interval.

### Functional Outcomes

At the last follow‐up, all patients were able to wear paired shoes and achieved satisfactory gait without any aid. ROMs of the injured ankle and hindfoot were significantly improved in aspects of ankle dorsal extension (22.0 ± 2.2° preoperatively, 38.8 ± 2.4° at last follow‐up, *p* < 0.001), plantar flexion (27.2 ± 1.8° preoperatively, 43.5 ± 3.3° at last follow‐up, *p* < 0.001), eversion (6.0 ± 2.8° preoperatively, 14.0 ± 3.5° at last follow‐up, *p* = 0.001), inversion (12.3 ± 2.3° preoperatively, 23.2 ± 1.8° at last follow‐up, *p* < 0.001), and HL (1.3 ± 1.2 cm preoperatively, 5.8 ± 0.8 cm at last follow‐up, *p* < 0.001), with minimal CC loss (31.0 ± 3.2 cm of the healthy limb preoperatively, 31.0 ± 2.4 cm of the injured limb at last follow‐up, *p* = 0.999), indicating little calf muscle wasting of the injured limb (Table 2).

**TABLE 2:** Clinical and radiological outcomes.

	CC (cm)	DE (°)	PF (°)	Ev (°)	Inv (°)	HL (cm)	TCA (°)	CIA (°)	MDA (°)	ALA (°)	VAS (point)
Preoperative (CC, healthy limb)	31.0 ± 3.2	22.0 ± 2.2	27.2 ± 1.8	6.0 ± 2.8	12.3 ± 2.3	1.3 ± 1.2	12.0 ± 2.1	20.7 ± 1.6	15.9 ± 0.8	136.5 ± 2.9	50.8 ± 5.9
Last interview (CC, injured limb)	31.0 ± 2.4	38.8 ± 2.4	43.5 ± 3.3	14.0 ± 3.5	23.2 ± 1.8	5.8 ± 0.8	4.5 ± 0.4	27.6 ± 1.0	22.8 ± 1.0	126.0 ± 0.5	91.5 ± 2.7
*t* value	0.00	13.19	11.61	6.93	13.67	8.00	10.23	9.50	31.07	9.14	14.16
*p* value	0.999	< 0.001	<0.001	0.001	<0.001	<0.001	<0.001	<0.001	<0.001	<0.001	<0.001

Abbreviations: ALA, angle of longitudinal arch; CC, calf circumference; CIA, calcaneus inclination angle; DE, dorsal extension of ankle; Ev, ankle eversion; HL, heel lift; Inv, ankle inversion; MDA, metatarsal distal angle; PF, plantar flexion of ankle; TCA, tibio‐calcaneal angle; VAS, visual analogue scale.

### Fluoroscopic Outcomes

Foot and ankle alignment angles, including TCA (12.0° ± 2.1° preoperatively, 4.5° ± 0.4° at last follow‐up, *p* < 0.001, normal, less than 6° eversion), CIA (20.7° ± 1.6° preoperatively, 27.6° ± 1.0° at last follow‐up, *p* < 0.001, normal, from 25° to 28°), MDA (15.9° ± 0.8° preoperatively, 22.8° ± 1.0° at last follow‐up, *p* < 0.001, normal, from 20° to 26°), and ALA (136.5° ± 2.9° preoperatively, 126.0° ± 0.5° at last follow‐up, *p* < 0.001, normal, from 125° to 130°), were all corrected according to radiography (Table 2).

### Scaled Evaluations

Subjective evaluation according to VAS (50.8 ± 5.9 points preoperatively, 91.5 ± 2.7 points at the last follow‐up, *p* < 0.001) was significantly improved, and patients were all satisfied with the eventual results (Table 2).

Detailed AOFAS data were shown in Table 3. The total AOFAS was significantly improved (36.0 ± 6.4 points preoperatively, 92.7 ± 8.5 points at the last follow‐up, *p* < 0.001), and there were 3 excellent and 3 good results, respectively. The mental status of all patients was within the normal range, but mild insomnia was observed right after external fixation, while anxiety was slightly reduced 2 months after internal fixation was removed (Table 4).

**TABLE 3:** Detailed AOFAS evaluated preoperatively and at last interview.^a^

Case (preoperative/last interview)	Pain	ALSR	MWDB	Walking surfaces	Gait abnormality	Sagital motion	Hindfoot motion	AHS	Alignment	Total
1	30/40	0/10	0/5	0/5	4/8	8/8	3/6	0/8	0/10	45/100
2	20/30	0/10	0/5	0/5	0/8	8/8	3/6	0/8	0/10	31/90
3	30/30	0/7	2/4	0/3	0/8	8/8	3/6	0/8	0/10	43/84
4	20/40	0/10	0/5	0/5	4/8	8/8	3/6	0/8	0/10	35/100
5	20/40	0/10	0/5	0/5	0/8	8/8	3/6	0/8	0/10	31/100
6	20/30	0/7	0/4	0/5	0/4	8/8	3/6	0/8	0/10	31/82
Preoperative	23.3 ± 5.2	0.0 ± 0.0	0.3 ± 0.8	0.0 ± 0.0	1.3 ± 2.1	8.0 ± 8.0	3.0 ± 0.0	0.0 ± 0.0	0.0 ± 0.0	36.0 ± 6.4
Last interview	35.0 ± 5.5	9 ± 1.5	4.7 ± 0.5	4.7 ± 0.8	7.3 ± 1.6	8.0 ± 8.0	6.0 ± 0.0	8.0 ± 0.0	10.0 ± 0.0	92.7 ± 8.5
*t* value	3.80	14.23	8.77	6.50	6.71	/	/	/	/	13.78
*p* value	<0.001	<0.001	<0.001	<0.001	<0.001	/	/	/	/	<0.001

^a^ AHS, Ankle‐hindfoot stability; ALSR, activity limitations and support requirement; AOFAS, American Orthopedic Foot and Ankle Score; MWDB, maximum walking distance, blocks.

**TABLE 4:** Psychological status assessed with SCL‐90‐R questionnaire.

Case	Phobia T1/T2/T3/T4	Anxiety T1/T2/T3/T4	Depression T1/T2/T3/T4	Somatization T1/T2/T3/T4	Obsessive‐compulsive T1/T2/T3/T4	Sensitivity T1/T2/T3/T4	Hostility T1/T2/T3/T4	Insomnia T1/T2/T3/T4	Psychoneuroticism T1/T2/T3/T4
1	1.1/1/1/1	1.3/1.4/1.1/1	1.3/1.2/1.1/1.1	1.2/1.2/1.1/1	1/1/1/1	1.2/1.2/1.1/1.1	1/1/1/1	1/1.2/1/1	1/1.2/1/1
2	1/1/1/1	1.2/1.2/1.1/1	1/1.1/1.1/1	1/1/1/1	1/1/1/1	1/1.1/1/1	1/1/1/1	1.1/1.2/1/1	1/1/1/1
3	1.1/1.1/1/1	1.2/1.2/1.2/1	1.1/1.1/1.1/1.1	1/1/1/1	1/1/1/1	1.1/1.2/1/1	1/1.1/1/1	1/1.2/1.1/1	1/1/1/1
4	1/1.1/1/1	1.2/1.2/1.2/1.2	1.1/1.1/1.1/1.1	1/1/1/1	1/1/1/1	1.1/1.1/1.1/1.1	1/1/1/1	1.1/1.2/1.1/1	1/1/1/1
5	1/1.1/1.1/1	1.1/1.2/1.1/1.1	1.3/1.4/1.3/1.2	1.1/1.2/1.1/1.1	1/1/1/1	1.1/1.2/1.2/1.1	1.1/1.1/1.1/1.1	1/1.2/1.1/1	1.1/1.1/1/1.1
6	1/1.1/1.1/1	1.1/1.2/1.2/1	1.2/1.3/1.1/1	1/1/1/1	1/1/1/1	1.1/1.1/1.1/1.1	1/1/1/1	1.2/1.2/1.2/1.1	1/1/1/1
Mean	1/1.1/1/1	1.2/1.2/1.2/1.1	1.2/1.2/1.1/1.1	1.1/1.1/1/1	1/1/1/1	1.1/1.2/1.1/1.1	1/1/1/1	1.1/1.2/1.1/1	1/1.1/1/1
*p*1/*p*2/*p*3	0.363/1.000/0.175	0.076/0.465/0.043	0.363/0.203/0.093	0.363/0.363/0.363	−/−/−	0.076/0.611/0.175	0.363/−/−	0.010/0.611/0.175	0.363/0.363/−

Abbreviations: *p*1, *p* value between T1 and T2; *p*2, *p* value between T1 and T3; *p*3, *p* value between T1 and T4; SCL‐90‐R, Symptom Checklist‐90‐Revised; T1, 2 days before external fixation; T2, 10 days after external fixation; T3, the day external fixation replaced by internal fixation; T4, 2 months after internal fixation removed.

### Typical Case

**FIGURE 2:**
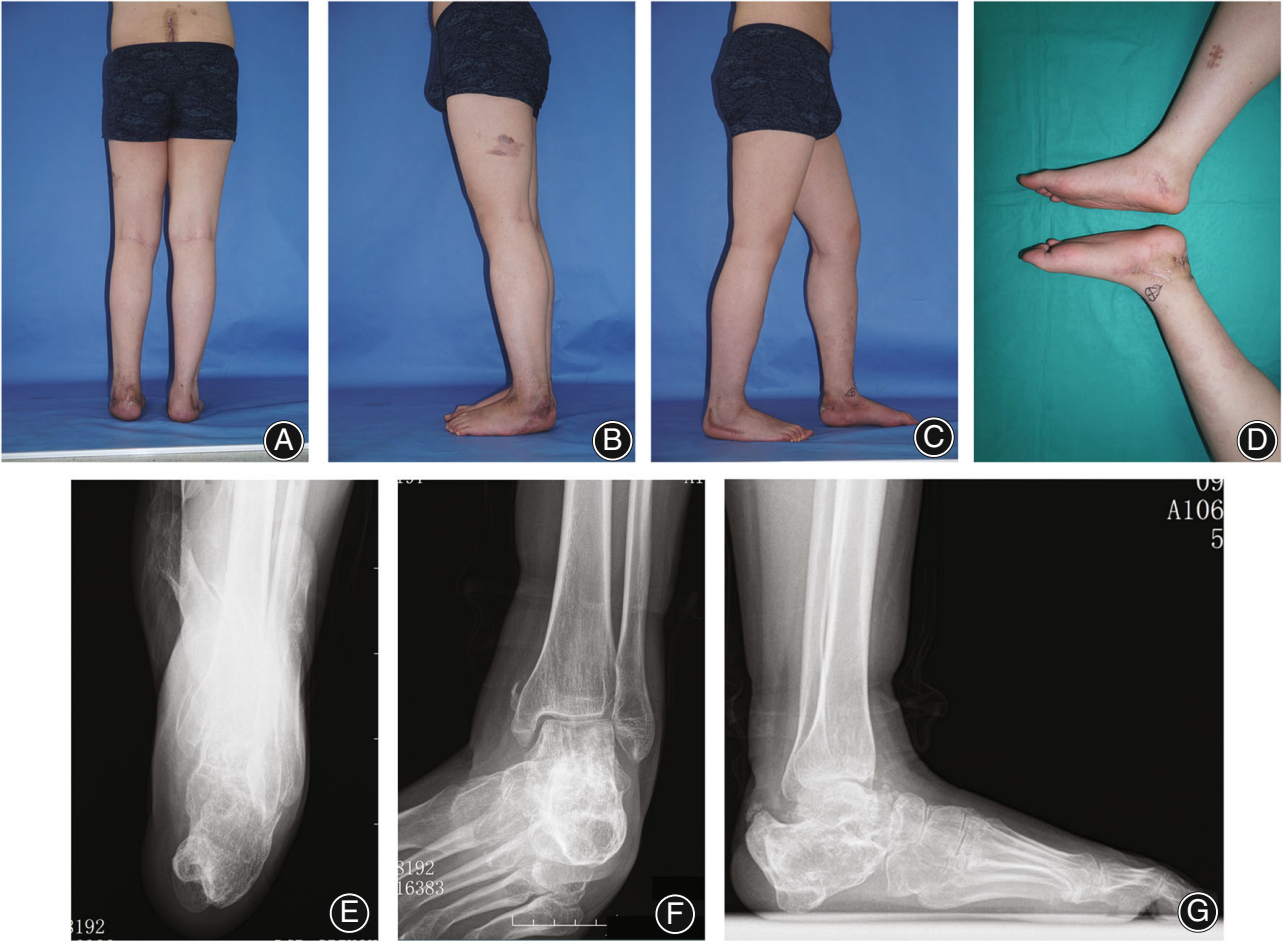
Preoperative appearance and radiography of the typical case. (A–D) The affected foot and ankle were in poor appearance with obvious malleolar varus and hindfoot deficiency. (E–G) Fluoroscopy showed a defect calcaneus with abnormal calcaneal interface angle (CIA), angle of a longitudinal arch (ALA), metatarsal declination angle (MDA), and tibio‐calcaneal angle (TCA).

**FIGURE 3:**
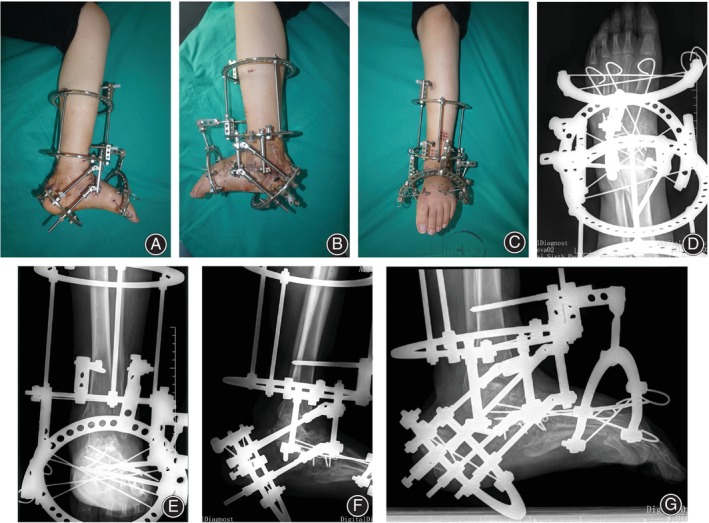
Triple arthrodesis was performed, and the Ilizarov apparatus was applied for the gradual lengthening of the remnant of the calcaneus. (A–C) Appearance during the lengthening period. (D–G) Fluoroscopy showed osteointegration after arthrodesis and detected the callus formation at the distraction zone.

**FIGURE 4:**
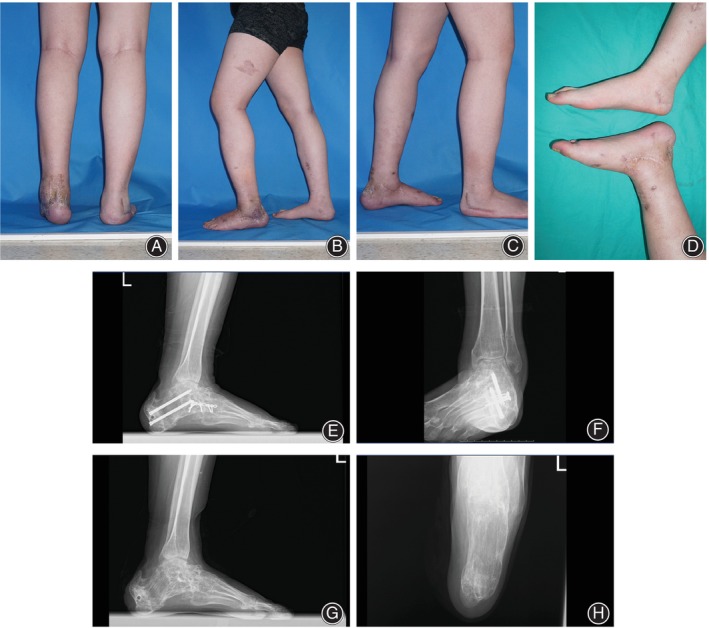
Postoperative appearance and radiography of the typical case. (A–D) The appearance of the affected foot and ankle was improved with malleolar varus and hindfoot deficiency corrected. (E–H) Fluoroscopy showed that calcaneal interface angle (CIA), angle of a longitudinal arch (ALA), metatarsal declination angle (MDA), and tibio‐calcaneal angle (TCA), as well as the length of the calcaneus, were restored.

**FIGURE 5:**
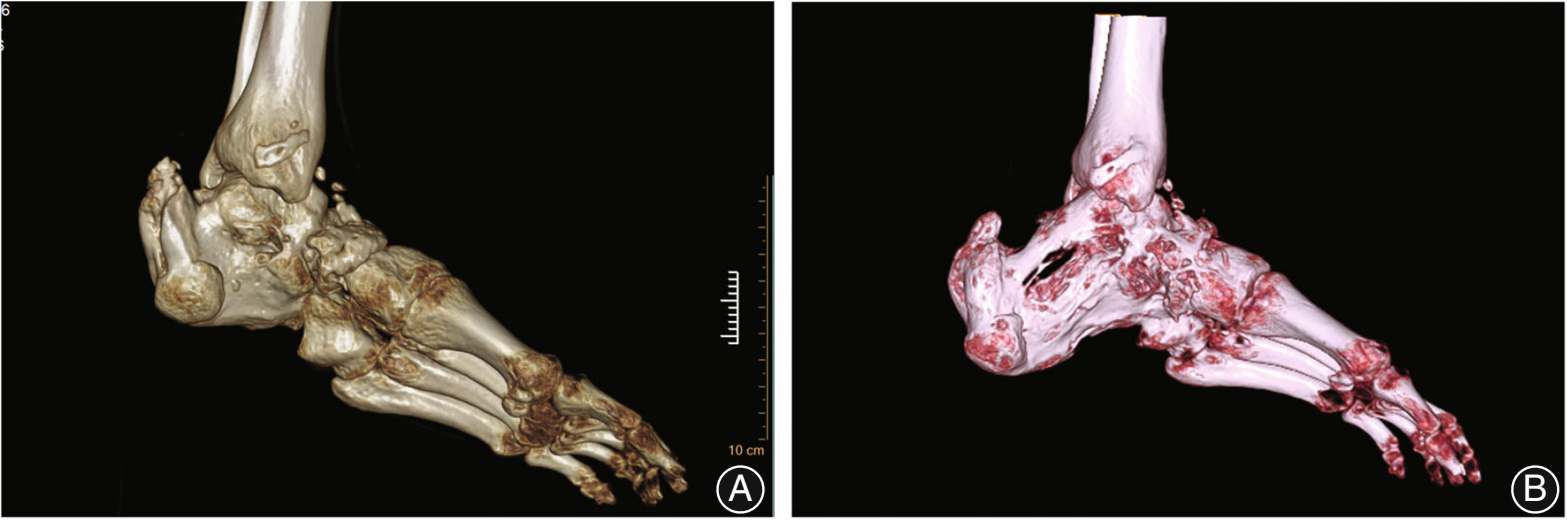
Three‐dimensional reconstruction of the affected foot based on computed tomography. (A) Preoperative appearance of the affected foot after three‐dimensional reconstruction. (B) Alignment of the involved ankle and foot was ultimately reconstructed.

The managing process of case 1 was shown in Figures 2A–G, 3A–G, and 4A–H. Alignment of the involved ankle and foot was ultimately reconstructed (Figure 5).

## Discussion

In this study, we introduced the experience of tarsal bone fusion and calcaneus gradual lengthening in treating traumatic massive calcaneus defect. All patients restored the normal length of the calcaneus after the elongation compared with the healthy side. Longitudinal foot arch, as well as hindfoot stability, was also well reconstructed after tarsal bone fusion. Although the range of ankle eversion and inversion was somehow sacrificed, the overall function of the injured limb was definitely improved according to the AOFAS comparison. The mental status of each patient remained in the normal range during the period of external fixation, except for temporary mild insomnia. All six patients in our study ultimately achieved good to excellent outcomes with no major complications.

### Literature Review

Calcaneus defect can significantly decrease the quality of life of the patients because of the disruption of normal biomechanics and gait of the injured limb.^9^ However, treatment options for such deformity have not been well‐defined yet because of its rarity.

Preservation of such injuries remains controversial. Although primary amputation offers early rehabilitation as well as reduced hospitalization, amputation is still definitely a tough decision for patients with lifelong disability and prosthesis maintenance fees. Recent decades have witnessed novel solutions for complex hindfoot defects with various success rates, which included osteofasciocutaneous flap surgery,^10^, ^11^, ^12^, ^13^, ^14^, ^15^, ^16^ allograft with a combination of autologous bone graft or bioactive adjuncts to induce osteogenesis,^17^, ^18^, ^19^, ^20^, ^21^, ^22^ and alloy substitution.^3^, ^23^, ^24^ Drawbacks of these approaches lie in donor site morbidity, difficulties in microsurgical technique, rejection reaction, long‐term bone absorption and fracture, material fatigue, and infection.

Ilizarov method has been proven quite effective in the treatment of limb deformities, including deficiency, angulation, and rotation. However, only a few surgeons have introduced their strategies based on the Ilizarov technique for limb salvage when dealing with challenging calcaneal defects. Wardak et al.^25^ first demonstrated the efficacy of Ilizarov ring fixators in treating hindfoot defects due to battlefield injury and traffic accidents by malleolectomy, tibial osteotomy and elongation, namely, calcanisation of the tibia, and tarsal joint fusion. Most of their patients had satisfactory results as they achieved stable limbs, walked without aids, and took only minor to moderate modifications to their daily activities, whereas most of them had difficulty in walking over uneven surfaces because the surgical procedure involved both malleolar arthrodesis and tarsal joint fusion, whether these joints were intact or not. Another study applied first‐stage fusion between the tibia and the talus with gradual lengthening of the tibia to create enough height of the hindfoot in patients with a complete absence of the calcaneus but intact talus.^26^ A second‐stage posterior transport of the reconstructed hindfoot was then added to mimic the foot arches.^26^ Other researchers attempted to perform elongation of the calcaneus, if it was the only defect tarsal bone, to reconstruct the alignment of the hindfoot and to preserve ROM of the affected ankle. Jielile et al.^27^ performed staged gradual elongation of the defect calcaneus in four pediatric patients caused by motorcycle spoke injury after debridement and flap coverage. They pointed out that the osteotomy must be performed close to sideways to obtain an adequate posterior calcaneal segment for transplantation. This was also noticed by Wang et al.^4^ when they carried out calcaneus gradual lengthening in adults with traumatic calcaneal defect, and they indicated that simple calcaneal gradual elongation was safe and effective in patients whose proportion of the calcaneal tuberosity defect should be within 10%–35% of the volume of the healthy calcaneus. They believed that a defect less than 10% could be mended up with immediate lengthening, while a defect larger than 35% would result in insufficient space for stable fixation of the osteotomized segments, leaving a blank for the application of Ilizarov technique in calcaneus reconstruction with a larger amount of loss.

### Pearls and Pitfalls

Taking advantage of both the Ilizarov technique and triple arthrodesis, we first realigned the hindfoot through osteotomy and tarsal bone fusion and then restored the length of the calcaneus as well as the ALA through gradual lengthening. As long as the posterior osteotomized segment is adequate for stable fixation and transplantation, the anterior residual of the osteotomized calcaneus can be fixed to adjacent tarsal and metatarsal bones through internal fixation.

Compared with all methods above, our calcaneus lengthening after the tarsal bone fusion technique can avoid microsurgical anatomy, prevent donor site morbidity, reduce foreign‐body rejection, and avert implant material fatigue. Although the Ilizarov technique guaranteed eventual reconstruction of the calcaneus and the heel via distraction histogenesis, prolonged external fixation time might lead to disuse osteoporosis and calf muscle atrophy of the injured limb due to the elongation model would hinder the hindfoot from weight‐bearing during lengthening and consolidation, according to previous studies applying such technique. Besides, after the removal of the external fixation, below‐knee casts were arranged in Wardak's trial for another 3 months to ensure consolidation, which significantly delayed the weight‐bearing rehabilitation of the affected limb.^25^ Hence, we also performed screw internal fixation to replace the external fixators as soon as an adequate amount of calcaneus length was obtained in order to shorten EFT and to get rehabilitation of the ankle as early as possible. The technique of internal fixation after external fixation has already been successfully used in limb discrepancy cases.^28^, ^29^ Screw fixation provided not only compression to the distracted callus, which had been verified to enhance neovasculogenesis and osteogenesis but also added stability to the fused tarsal joints. Furthermore, removal of the external fixation allowed early partial‐to‐full weight‐bearing of the foot and ankle to defend against osteoporosis and ankylosis.

However, whether triple arthrodesis should be performed in every patient suffering from calcaneus must be considered carefully. In our cases, the patients have already developed subtalar arthritis due to the trauma just before the distraction device installation, so we performed subtalar arthrodesis to give permanent relief to that joint pain and to stabilize the hindfoot. How to stabilize the proximal calcaneus during distraction histogenesis and simultaneous tibialis anterior tendon isometric contraction practice? We came up with the idea of cubitocalcaneal arthrodesis because the reconstructed tendon terminal may somehow move the proximal calcaneus segment and interrupt the healing of subtalar arthrodesis. Also, because the patient possessed forefoot and malleolar varus, additional cubitocalcaneal arthrodesis would then improve such deformities. For talonavicular arthrodesis, it was not necessary in every case, but we had to ensure that the medial longitudinal arch of the foot was in the expected position after the reconstruction.

Traumatic calcaneus defect has never been a simple elongation circumstance. Long periods of loss of calcaneus, along with soft tissue damage, will also create foot arch changes, arthritis, and muscle strength loss. After a rather long interval before reconstruction surgeries, these patients may then develop painful tarsal arthritis, imbalanced malleolar muscle strength, and abnormal arch angles due to compromised gait. Hence, when dealing with massive calcaneus defects through distraction histogenesis, surgeons should not only manage the obvious defect of calcaneus but also notice the symptoms and strength and stability problems that go beyond the calcaneus defect. Tarsal bone fusion would be a powerful solution for these symptoms and problems, as it allows adjusting the foot arch as well as to alleviate arthritic symptoms.

### Limitations

To our knowledge, this is the first study for calcaneus reconstruction with both distraction histogenesis and tarsal bone fusion. The study was an observational and narrative study to report the functional and mental outcomes of patients who suffered a long time from calcaneus defects and complicated hindfoot deformities. We have not held comparative groups in which patients received null, single, or double arthrodesis of the tarsal bones due to the rarity of prevalence, so it is hard to tell whether the triple arthrodesis is actually excessive or not. Although after a long period of struggling and adjustment against the initial trauma and calcaneus defect, the tarsal bones of these patients certainly would develop deformities that require correction, including osteotomy and arthrodesis.

### Prospects of Clinical Application

Occasional high‐energy traumas are still threatening the integrity of the hindfoot of those who are engaged. Our technique would be an alternative approach for hindfoot reconstruction in which circumstances the calcaneus is partially lost acutely or chronically due to reiterated debridement. The most important precondition for the application of this technique is that the talocalcaneal joint line of the involved hindfoot should be intact to indicate calcaneus gradual elongation rather than calcanisation of the tibia. With a deeper understanding of foot and ankle alignment, this lengthening plus arthrodesis technique will eventually be a powerful tool for massive partial calcaneus defect reconstruction.

## Conclusion

The Ilizarov technique remains a surprising and preferable option for calcaneus reconstruction, with a great amount of loss once combined with tarsal bone fusion. Apart from calcaneus elongation, tarsal bone fusion is somehow necessary to reinforce the proximal segment of the distracted calcaneus for creating a larger distraction callus, correcting concomitant foot deformities, and enhancing hindfoot stability. It is necessary to choose flexibly when tarsal bones should be fused. The function of the injured foot and ankle can be satisfactorily restored using these techniques. Further studies may focus on the biomechanics and long‐term outcomes of this composite of strategies.

## Author Contributions

All authors contributed to the creation of this article. Rui Zhang and Xiangyun Yao contributed equally to this article as co‐first authors. The authors contributed separately as follows. Concept/idea/research design: Hongjiang Ruan and Qinglin Kang. Acquisition of data: Rui Zhang and Xiaoyu Wang. Analysis and interpretation of data: Xiangyun Yao and Xu Zheng. Writing/review/editing of manuscript: Rui Zhang and Xiangyun Yao. Final approval of the manuscript: Hongjiang Ruan and Qinglin Kang. Acquisition of funding: Qinglin Kang. Providing facilities/equipment: Hongjiang Ruan. Providing subjects: Qinglin Kang and Haoran Mu. Identify the author (“guarantor”) who takes responsibility for the integrity of the work as a whole, from inception to published work: Qinglin Kang.

## Conflicts of Interest

All authors claim no financial disclosure or any conflict of interest.

## Name of the Public Trials Registry and the Registration Number

Chinese Clinical Trial Registry; Registration Number: ChiCTR2300072615.
